# Structural and Technological Approach to Reveal the Role of the Lipid Phase in the Formation of Soy Emulsion Gels with Chia Oil

**DOI:** 10.3390/gels7020048

**Published:** 2021-04-20

**Authors:** Ana M. Herrero, Claudia Ruiz-Capillas

**Affiliations:** Institute of Food Science, Technology and Nutrition (ICTAN-CSIC), José Antonio Novais 10, 28040 Madrid, Spain; claudia@ictan.csic.es

**Keywords:** soy protein, olive oil, emulsion gel, gel, protein structure, Raman spectroscopy, technological properties

## Abstract

Considerable attention has been paid to emulsion gels (EGs) in recent years due to their interesting applications in food. The aim of this work is to shed light on the role played by chia oil in the technological and structural properties of EGs made from soy protein isolates (SPI) and alginate. Two systems were studied: oil-free SPI gels (SPI/G) and the corresponding SPI EGs (SPI/EG) that contain chia oil. The proximate composition, technological properties (syneresis, pH, color and texture) and structural properties using Raman spectroscopy were determined for SPI/G and SPI/EG. No noticeable (*p* > 0.05) syneresis was observed in either sample. The pH values were similar (*p* > 0.05) for SPI/G and SPI/EG, but their texture and color differed significantly depending on the presence of chia oil. SPI/EG featured significantly lower redness and more lightness and yellowness and exhibited greater puncture and gel strengths than SPI/G. Raman spectroscopy revealed significant changes in the protein secondary structure, i.e., higher (*p* < 0.05) α-helix and lower (*p* < 0.05) β-sheet, turn and unordered structures, after the incorporation of chia oil to form the corresponding SPI/EG. Apparently, there is a correlation between these structural changes and the textural modifications observed.

## 1. Introduction

Notable advances have been made in recent decades in the design and preparation of emulsion gels (EGs) with the benefits of the technological/functional properties of both emulsions and gels. These EGs have novel applications in food and have proven to be potentially useful in a wide range of foods such as dairy and meat products [1,2,3,4,5,6].

Basically, EGs are complex colloidal lipid materials in which emulsion droplets and gels (hydrogels) coexist [1]. Different EGs with different structure–property relationships and applications can be obtained depending on the preparation method used [4]. EGs are prepared based on the formation of an emulsion by homogenizing an oil in an aqueous solution containing a hydrophilic emulsifier, ultimately forming solid structures by means of gelling processes [1]. Lipid particles (emulsion droplets) become stabilized as they are trapped inside the hydrogel [1,7]. In any case, gelling can be induced in several different ways, the following being the most noteworthy: by heating, by acidification, by adding divalent cations (Ca^2+^) or enzymatically [1].

As for the components that make up EGs, one of the most frequently used emulsifiers is the soy protein, specifically the soy protein isolate (SPI), as it is an excellent water and fat binder with both emulsifying and emulsion-stabilizing properties [8,9]. If a vegetable or marine oil with a healthy fatty acid profile such as olive, linseed or chia is used, the emulsion formed would offer interesting advantages [2,6]. Gelling processes that use cold-set binding agents merit special consideration as they give rise to cold-set EGs which may be more appropriate in delivering thermolabile compounds. Alginate stands out among the compounds used to induce cold gelling as it forms gels in the presence of calcium salt [10,11]. These gels are made up of polymeric molecules cross-linked to form a three-dimensional macromolecular network containing a large fraction of water within its mechanically rigid structure. In addition to functioning as gelling agents, they also exhibit emulsifying, stabilizing and other properties [12,13].

Preparation of these EGs renders lipid materials with suitable technological and nutritional properties (adjusted to health recommendations), provided that the appropriate ingredients for both purposes are used [2,5,6]. Under these conditions, in addition to their components and technological characteristics, it could also be necessary to take structural aspects into account to globally address their stability since the interactions between their components and structural state could affect the mechanical properties of EGs [1,14,15,16,17] and compromise their use in food products. In this field of research, Raman spectroscopy could play a vital role in providing the relevant structural information about the formation of EGs.

Raman spectroscopy is a direct technique which has proven useful in simultaneously providing structural information about the different components (lipids, proteins, etc.) used to prepare EGs [17,18,19,20,21,22]. Some authors have already proven the potential of this spectroscopic technique in studying the structural changes taking place when emulsions are formed using model systems [19,22] and specific EGs [16,17]. Gel matrix and emulsion droplet structures and interactions between the two can impact EG structures and hence their mechanical properties and stability [3,4]. Emulsion droplet structures are especially affected by the oil phase (type, oil content and oil droplet size, among other factors) [4]. Hence, more research is needed on the role played by the oil phase in the technological and structural properties of EG formation.

The objective of this work was, therefore, to evaluate the changes in the technological properties (color, texture, etc.) and structural characteristics caused by the oil phase in the formation of SPI EGs formulated with chia oil. To that end, we compared two systems with similar gel matrices, but one that was oil-free (SPI gels) and one with chia oil (SPI EGs), to understand the role of the lipid phase in the formation of soy EGs. Raman spectroscopy was chosen as the optimal method since it is a non-destructive analytical tool with great potential to provide structural information on EGs.

## 2. Results and Discussion

Two different sample types were studied: oil-free SPI gels (SPI/G) and SPI emulsion gel with chia oil (SPI/EG). The formulations used to prepare these samples are described in Table 1.

### 2.1. Proximate Composition

Moisture contents (%) were significantly different in SPI/G (73.9 ± 0.27^a^) and SPI/EG (33.4 ± 0.51^b^) according to the formulation (Table 1). SPI/EG had a higher (*p* < 0.05) ash content (1.6 ± 0.03^a^%) than SPI/G (1.02 ± 0.06^b^%), likely due to the mineral content of chia oil [23]. A similar protein content was found in SPI/G (21.50 ± 0.21^a^) and SPI/EG (19.83 ± 0.16^a^). The fat content in SPI/EG was 38.9 ± 1.28%. Chia has relatively low levels of saturated fatty acids and its fatty acid profile contributes about 34 g of polyunsaturated fatty acids (PUFA)/100 g oil (22.8–26 g α-linolenic/100 g oil), therefore giving these EGs health benefits [24]. The PUFA content of this SPI/EG could be delivered to an end product such as a meat product in which these EGs were incorporated to replace animal fat.

### 2.2. Syneresis and pH Values

The water and fat binding properties of the two different samples (SPI/G and SPI/EG) were positive with no apparent syneresis following preparation. Similar findings have been described for other EGs and oil-free gels [16,25,26,27].

Table 2 shows that sample pH values were similar (*p* > 0.05) for SPI/G and SPI/EG and were not affected (*p* > 0.05) by chia oil. These pH values were within the ranges reported for other EGs prepared with soy protein [16,28,29] and oil-free gels with similar characteristics [25].

Considering that their syneresis and pH values are similar to those of pork back fat [27], SPI/G and SPI/EG could be used as animal fat replacers to create healthier meat products with improved lipid content.

### 2.3. Color Measurement

The original color of the SPI gel changed significantly with the addition of chia oil (Table 2). Lightness increased (*p* < 0.05) when chia oil was incorporated (SPI/EG) (Table 2). The oil in SPI/EG also lowered (*p* < 0.05) redness and accentuated (*p* < 0.05) yellowness (Table 2). Findings were similar for a* and b* values when the olive oil content was increased in oat emulsion gels and when these EGs were compared with oil-free oat gels [25]. The increase in b* could be attributable to the yellowish-green hue of chia oil. It is worth noting that the vegetable oil color is associated with the total pigment content and the presence of carotenoids in chia seed oils [30,31].

### 2.4. Penetration Test

Both SPI/G and SPI/EG exhibited a clear breaking point in the force–deformation curves, indicating that they have a typical gel structure. Chia oil impacted the texture in the samples, SPI/EG exhibiting higher puncture force (PF) and gel strength (GS) values (*p* < 0.05) (Table 2). The dispersed particles comprising EGs can be classified as active or inactive depending on their contribution to the matrix network. Inactive fillers exhibit little chemical affinity for the matrix network and hence do not strengthen the gel, while active fillers interact with the gel matrix, resulting in increased gel strength [1,32]. In view of these facts, the greater gel strength observed in SPI/EG compared to SPI/G (Table 2) could be due to the fact that the droplet chia oil in SPI/EG acts as an active filler reinforcing the gel matrix. Other authors have also found this in the case of EGs produced with both proteins and polysaccharides filled with solid lipid particles and they found that the lipid particles could couple fatty acids of higher melting points into emulsion gels and enhanced the GS [3,33]. In these types of EGs, it was also found that the increased GS coinciding with the higher oil content means that the high volume of oil was performing as an active filler in EGs [3,33].

### 2.5. FT-Raman Spectroscopic Analysis

#### Protein Secondary Structure 

**Figure 1:**
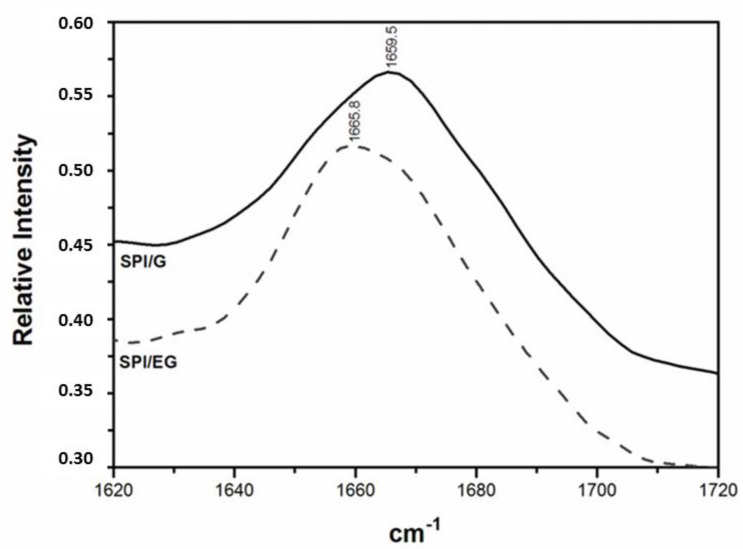
Raman spectra in the 1620–1720 cm^−1^ region of oil-free SPI gels (SPI/G) and SPI emulsion gels with chia oil (SPI/EG). See Table 1 for sample formulation.

The Raman spectral region around 1600–1700-cm^−1^ is the one most often used to extract information on the protein secondary structure [17,18,20]. This spectral region is dominated by an amide I band (Figure 1), a strong band that primarily represents the C=O stretching vibrations of the amide groups (coupled to in-plane bending of the N–H bonds and stretching of the C–N bonds) [17,18,20]. Figure 1 compares SPI/G and SPI/EG, where a shift can be observed in the maximum absorption frequency from 1659.5 to 1665.8 cm^−1^. This indicates changes in the secondary protein structure when chia oil is incorporated into SPI/G and the corresponding emulsion gel SPI/EG is formed [34]. Other authors using infrared spectroscopy have shown a similar shift in the amide I band to higher frequencies when oil-in-water emulsions were formed [34,35]. This phenomenon has been attributed to structural changes in secondary proteins which enrich the α-helix structure in emulsions after protein adsorption in oil/water interfaces [35].

**Figure 2:**
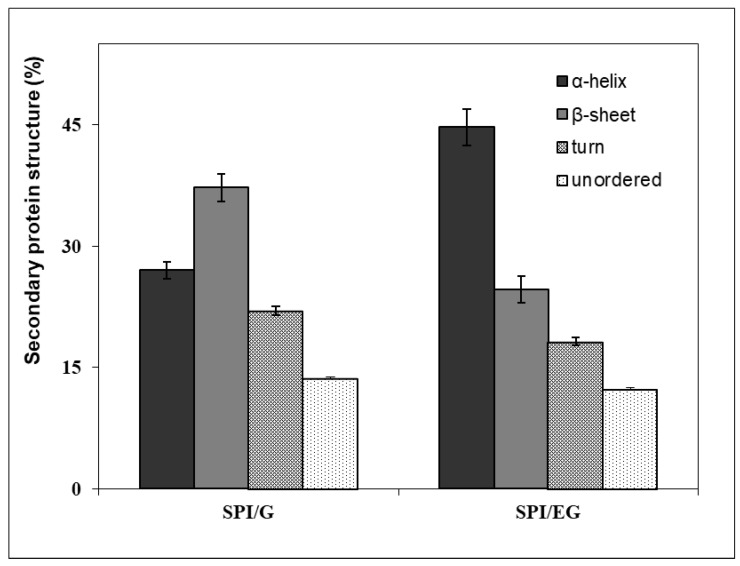
Percentages of α-helix, β-sheet, turn and unordered protein structures of oil-free SPI gels (SPI/G) and SPI emulsion gels with chia oil (SPI/EG). See Table 1 for sample formulation.

These secondary protein structural changes were confirmed by means of a quantitative analysis of the amide I band profile from which the α-helix, β-sheet, turn and unordered structure were obtained [18,36]. To that end, as described in the literature, the water spectrum was first subtracted eliminating the 2125 cm^−1^ band of water using a subtraction factor until the intensity of this band was no longer visible (18–36). Figure 2 shows the comparison of the protein secondary structure percentages obtained for SPI/G and SPI/EG. There was a significant increase (*p* < 0.05) in α-helices along with a significant decrease (*p* < 0.05) in β-sheet, turn and unordered structures after chia oil was incorporated into the gel matrix to form the corresponding emulsion gel (SPI/EG) (Figure 2). The α-helix structure is a higher-order helical structure, more stable in general than β-sheet since it is stabilized by the regular formation of hydrogen bonds parallel to the axis of the helix. Other researchers have observed interactions between chia oil ester molecules and proteins upon formation of chia oil EGs [16] that could be related to the structural changes in secondary proteins observed in SPI/EG (Figure 2) due to the effect that chia oil has on the gel network.

The differences in the secondary protein structure between SPI/G and SPI/EG due to the addition of chia oil (Figure 2) appear to have a decisive impact on the texture of these samples (Table 2). Therefore, it would be reasonable to assume that the significant increase in α-helices and the concomitant decrease in β-sheet, turn and unordered structures in SPI/EG are related to the greater PF and GS observed following the incorporation of chia oil in these EGs. Similar relationships between specific structural characteristics and certain textural properties of EGs have been reported in the literature [15,26,34,37]. Comparable inputs could be obtained with other edible oils with a composition and functionality similar to chia oil, but further work would be required to confirm this assumption.

## 3. Conclusions

By comparing SPI oil-free emulsion gels (SPI/G) and their corresponding SPI EGs prepared with chia oil (SPI/EG), we were able to study the role played by the lipid phase in the formation of these EGs and how it affects certain technological and structural properties. The incorporation of chia in the gel matrix mostly affects technological properties such as the texture and color. Raman spectroscopy has proven to be a powerful tool to provide direct information about the protein secondary structural changes that occur by chia oil in the formation of EGs. This spectroscopic technique revealed a significant increase in alfa helix structures and a decrease in β-sheet, turn and unordered structures which could be related to a specific textural behavior when chia oil is involved in the formation of the EG gel matrix. This texture–structure relationship could help to understand the global role of chia oil in EGs.

## 4. Materials and Methods

### 4.1. Sample Preparation

Two systems with similar gel matrices, one with chia oil (SPI/EG) and other oil-free (SPI/G), were prepared to understand the role of the lipid phase in the formation of soy EGs. These samples were prepared with soy protein isolate (SPI): (a) oil-free SPI gel (SPI/G) and (b) SPI emulsion gel (SPI/EG), both prepared with 23% SPI, 74.25 and 34.25% water for SPI/G and SPI/EG, respectively, and an alginate-based gelling agent (1% sodium alginate, 1% CaSO_4_ and 0.75% tetra-sodium pyrophosphate). SPI/EG also included 40% chia oil (Table 1).

The ingredients used to prepare SPI/G and SPI/EG were procured from different suppliers: SPI (90% protein content) from Manuel Riesgo SA (Madrid, Spain); sodium alginate (90% carbohydrate content) from Tradissimo (TRADES S.A., Barcelona, Spain); CaSO_4_ and tetra-sodium pyrophosphate anhydrous from Panreac Química, S.A. (Madrid, Spain); and unrefined chia oil from Primaria Premium Raw Materials, S.L (Valencia, Spain). The chia oil contained a total of approximately 85 g of PUFA/100 g oil (57–65 g α-linolenic/100 g oil).

**Figure 3:**
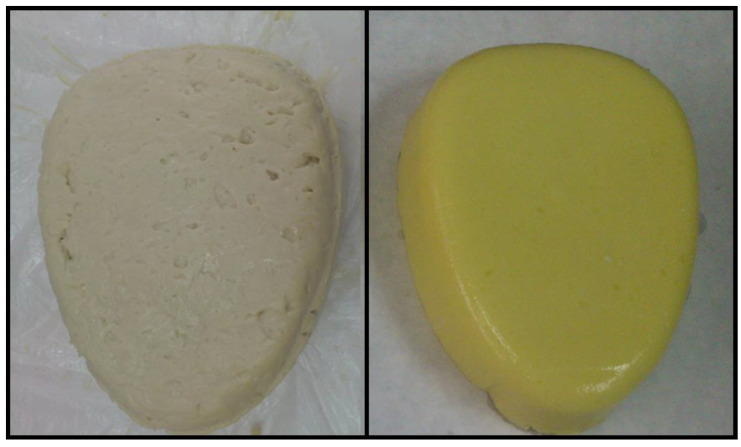
Representative appearance of oil-free SPI gel (SPI/G, **left**) and SPI emulsion gel (SPI/EG, **right**). For sample denomination see Table 1.

Samples (SPI/G and SPI/EG) were prepared at room temperature by mixing, in a homogenizer (Thermomix TM 31, VorwerkEspaña M.S.L., S.C, Madrid, Spain), SPI with the corresponding amount of water for each type of sample (Table 1) for 30 s at approximately 5600 rpm. The alginate-based gelling agent was then added, and the entire mixture blended again (15 s at approximately 5600 rpm). Chia oil was gradually added to the SPI/EG mixture only (Table 1) while the blender was in operation (5600 rpm). Lastly, each sample type was placed in a metal container under pressure (maximum pressure to avoid cavities or air bubbles in the mixture) and refrigerated at 2 °C for 20 h to allow for gelification. Samples were then removed from their metal containers and analyzed. Each sample type was prepared in triplicate. Figure 3 shows the final SPI/G and SPI/EG gels obtained.

### 4.2. Proximate Composition Analysis

Sample moisture content was determined by weight difference between raw samples and samples after treatment in an oven at 100 °C for 24 h [38]. Ash content was obtained by weight difference between raw samples and samples after treatment in a muffle furnace at 550 °C for 24 h [38]. Protein content was measured using 1 g of sample with a LECO FP-2000 Nitrogen Determinator (Leco Corporation, St. Joseph, MI, USA). Fat content was evaluated as g/100 g product [39]. Briefly, 10 g of sample was homogenized with a methanol/chloroform mixture successively in several steps to extract the fat from the samples. Subsequently, the phases obtained were separated using a decantation procedure, collecting the chloroformic part in which the extracted fat is found [39]. All determinations were performed in triplicate.

### 4.3. Syneresis and pH Values

Syneresis was determined in triplicate as the amount of exudate in samples, including fat and water loss, by weight difference (%) between the initial weight of the mixture put in the container and the final sample weight obtained after removing the sample from the container.

pH was measured in triplicate using an 827 Metrohm pH-meter (MetrohmAG, Herisau, Switzerland) on sample homogenates in distilled water at a ratio of 1:10 *w*/*v*.

### 4.4. Color Measurement

Color (CIE-LAB tristimulus values, lightness, L*; redness, a*; and yellowness, b*) was evaluated on a Chroma Meter CR-400 (Konica Minolta Business Technologies, Inc., Tokyo, Japan). Determinations were carried out on cross-sections of sample. Ten determinations were performed from each sample type.

### 4.5. Penetration Test

Penetration tests were conducted at approximately 22 °C with a TA-XT.plus Texture Analyzer (Texture Technologies Corp., Scarsdale, Westchester County, New York, NY, USA) along with the Texture Exponent program (Stable Micro Systems, London, UK) [34]. A 5 kg load cell was used, and analysis was performed with a 4 mm diameter cylindrical stainless-steel plunger at a velocity of 0.8 mm/s with force exerted at 10 mm. The following textural parameters for each sample were obtained from their force–deformation curve: (a) penetration force (PF, N) at the point of gel rupture and (b) gel strength (GS, Nmm), defined as the area enclosed by the force–deformation curve at the point of gel rupture. Six determinations per sample were carried out.

### 4.6. FT-Raman Spectroscopic Analysis

Portions of SPI/G and SPI/EG were placed in quartz cuvettes (ST-1/Q/10) (TEKNOKROMA, Barcelona, Spain) to fill them to a length of 1 cm. A total of 1500 scans were recorded for each sample. This procedure was carried out in triplicate, giving a total of 4500 scans per sample. These measurements were performed in triplicate for each sample type (SPI/G and SPI/EG). Spectra were excited with the 1064 nm Nd: YAG laser line and recorded on a Bruker RFS 100/S FT-spectrometer (Bruker, Karlsruhe, Germany). Scattered radiation was collected at 180° with respect to the source, and the frequency-dependent scattering of the Raman spectra produced by the spectrometer was corrected by multiplying point by point (mlaser/m)4. The optic effect on the spectrometer was corrected by using the Raman correction command included in the Opus 2.2 software (Bruker, Karlsruhe, Germany). Reported frequencies are accurate to ±0.5 cm^−1^ as deduced from frequency standards measured in the spectrometer. Raman spectra were resolved at 4 cm^−1^ resolution with a liquid nitrogen-cooled Ge detector. Samples were illuminated by laser power at 300 mW.

Raman spectra were analyzed using the Bruker Opus 2.2 and Grams/AI version 9.1 (Thermo Electron Corporation, Waltham, MA, USA) software. The Phe *ν*-ring band located near 1006 cm^−1^ was used as an internal standard to normalize the spectra as it has been reported to be insensitive to the micro-environment [18,20]. 

### 4.7. Statistical Analysis

One-way analysis of variance (ANOVA) was performed to calculate the statistical significance (*p* < 0.05) of the influence of sample composition on pH, color, penetration test parameters (PF and GS) and Raman spectroscopic data using the SPSS Statistics general linear model (GLM) procedure (v.22, IBM SPSS Inc., Chicago, IL, USA). Least square differences were used for comparison of mean values among formulations and Tukey’s HSD test to identify significant differences (*p* < 0.05) between samples. Data were expressed as mean values and standard error of the mean.

## Figures and Tables

**Table 1:** Formulation (%) of oil-free SPI gels (SPI/G) and SPI emulsion gels with chia oil (SPI/EG).

Samples *	SPI	Gelling Agent			Water	Chia Oil
		Sodium Alginate	CaSO_4_	Sodium Pyrophosphate		
SPI/G	23.25	1	1	0.75	74	
SPI/EG	23.25	1	1	0.75	34	40

* Oil-free soy protein isolate (SPI) gel (SPI/G) and SPI emulsion gel with chia oil (SPI/EG).

**Table 2:** pH values, color parameters ((L*) lightness, (a*) redness and (b*) yellowness) and penetration test parameters (puncture force (PF, N) and gel strength (GS, Nmm)) of oil-free SPI gels (SPI/G) and SPI emulsion gels with chia oil (SPI/EG).

Parameters	Samples *	
	SPI/G	SPI/EG
pH	7.26 ± 0.04 ^a^	7.31 ± 0.03 ^a^
L*	64.77 ± 0.87 ^b^	74.61 ± 0.81 ^a^
a*	3.14 ± 0.32 ^a^	1.52 ± 0.20 ^b^
b*	18.56 ± 0.41 ^b^	19.45 ± 0.23 ^a^
PF	1.21 ± 0.33 ^b^	1.87 ± 0.04 ^a^
GS	10.40 ± 0.86 ^b^	17.93 ± 0.66 ^a^

* See Table 1 for sample formulation. Means ± standard deviation. Different letters in the same row indicate significant differences (*p* < 0.05).

## Data Availability

Not applicable.

## References

[1] Dickinson E. (2012). Emulsion gels: The structuring of soft solids with protein-stabilized oil droplets. Food Hydrocoll..

[2] Jimenez-Colmenero F., Salcedo-Sandoval L., Bou R., Cofrades S., Herrero A.M., Ruiz-Capillas C. (2015). Novel applications of oil structuring methods as a strategy to improve the fat content of meat products. Trends Food Sci. Technol..

[3] Lu Y., Mao L., Hou Z., Miao S., Gao Y. (2019). Development of Emulsion Gels for the Delivery of Functional Food Ingredients: From Structure to Functionality. Food Eng. Rev..

[4] Lin D., Kelly A.L., Miao S. (2020). Preparation, structure-property relationships and applications of different emulsion gels: Bulk emulsion gels, emulsion gel particles, and fluid emulsion gels. Trends Food Sci. Technol..

[5] Paglarini C.D.S., Vidal V.A.S., Martini S., Cunha R.L., Pollonio M.A.R. (2020). Protein-based hydrogelled emulsions and their application as fat replacers in meat products: A review. Crit. Rev. Food Sci..

[6] Herrero A.M., Ruiz-Capillas C. (2021). Novel lipid materials based on gelling procedures as fat analogues in the development of healthier meat products. Curr. Opin. Food Sci..

[7] McClements D.J. (2012). Advances in fabrication of emulsions with enhanced functionality using structural design principles. Curr. Opin. Colloid Interface Sci..

[8] Matsumura Y., Sirison J., Ishi T., Matsumiya K. (2017). Soybean lipophilic proteins—Origin and functional properties as affected by interaction with storage proteins. Curr. Opin. Colloid Interface Sci..

[9] Tang C.H. (2017). Emulsifying properties of soy proteins: A critical review with emphasis on the role of conformational flexibility. Crit. Rev. Food Sci..

[10] Zhang J., Daubert C.R., Foegeding E. (2005). Fracture analysis of alginate gels. J. Food Sci..

[11] Roopa B.S., Bhattacharya S. (2010). Alginate gels: II. Stability at different processing conditions. J. Food Process Eng..

[12] Gaonkar A.G. (1991). Surface and interfacial activities and emulsion characteristics of some food hydrocolloids. Food Hydrocoll..

[13] Herrero A.M., Jimenez-Colmenero F., Ruiz-Capillas C., Raikos V., Ranawana V. (2019). Improving lipid content in muscle-based food: New strategies for developing fat replacers based on gelling processes using healthy edible oils. Reformulation as a Strategy for Developing Healthier Food Products.

[14] Dickinson E. (2013). Stabilising emulsion-based colloidal structures with mixed food ingredients. J. Sci. Food Agric..

[15] Pintado T., Ruiz-Capillas C., Jimenez-Colmenero F., Carmona P., Herrero A.M. (2015). Oil-in-water emulsion gels stabilized with chia (*Salvia hispanica* L.) and cold gelling agents: Technological and infrared spectroscopic characterization. Food Chem..

[16] Herrero A.M., Ruiz-Capillas C., Pintado T., Carmona P., Jimenez-Colmenero F. (2018). Elucidation of lipid structural characteristics of chia oil emulsion gels by Raman spectroscopy and their relationship with technological properties. Food Hydrocoll..

[17] Ruiz-Capillas C., Herrero A.M. (2021). Development of Meat Products with Healthier Lipid Content: Vibrational Spectroscopy. Foods.

[18] Herrero A.M. (2008). Raman spectroscopy for monitoring protein structure in muscle food systems. Crit. Rev. Food Sci..

[19] Howell N.K., Herman H., Li-Chan E.C.Y. (2001). Elucidation of protein-lipid interactions in a lysozyme-corn oil system by Fourier transform Raman spectroscopy. J. Agric. Food Chem..

[20] LiChan E.C.Y. (1996). The applications of Raman spectroscopy in food science. Trends Food Sci. Technol..

[21] Muik B., Lendl B., Molina-Díaz A., Ayora-Cañada M.J. (2003). Direct, reagent free determination of free fatty acid content in olive oil and olives by Fourier transform Raman spectrometry. Anal. Chim. Acta.

[22] Meng G., Chan J.C.K., Rousseau D., Li-Chan E.C.Y. (2005). Study of protein-lipid interactions at the bovine serum albumin/oil interface by Raman microspectroscopy. J. Agric. Food Chem..

[23] Capitani M.I., Nolasco S.M., Tomas M.C. (2016). Stability of oil-in-water (O/W) emulsions with chia (*Salvia hispanica* L.) mucilage. Food Hydrocoll..

[24] Ayerza R., Coates W. (2005). Ground chia seed and chia oil effects on plasma lipids and fatty acids in the rat. Nutr. Res..

[25] Pintado T., Herrero A.M., Jiménez-Colmenero F., Ruiz-Capillas C. (2016). Emulsion gels as potential fat replacers delivering β-glucan and healthy lipid content for food applications. J. Food Sci. Technol..

[26] Muñoz-González I., Merino-Álvarez E., Salvador M., Pintado T., Ruiz-Capillas C., Jiménez-Colmenero F., Herrero A.M. (2019). Chia (*Salvia hispanica* L.) a promising alternative for conventional and gelled emulsions: Technological and lipid structural characteristics. Gels.

[27] Jiménez-Colmenero F., Cofrades S., Herrero A.M., Fernández-Martín F., Rodríguez-Salas L., Ruiz-Capillas C. (2012). Konjac gel fat analogue for use in meat products: Comparison with pork fats. Food Hydrocoll..

[28] Jiménez-Colmenero F., Herrero A., Pintado T., Solas M.T., Ruiz-Capillas C. (2010). Influence of emulsified olive oil stabilizing system used for pork backfat replacement in frankfurters. Food Res. Int..

[29] Muñoz-González I., Ruiz-Capillas C., Salvador M., Herrero A.M. (2021). Emulsion gels as delivery systems for phenolic compounds: Nutritional, technological and structural properties. Food Chem..

[30] Imran M., Nadeem M., Manzoor M.F., Javed A., Ali Z., Akhtar M.N., Ali M., Hussain Y. (2016). Fatty acids characterization, oxidative perspectives and consumer acceptability of oil extracted from pre-treated chia (*Salvia hispanica* L.) seeds. Lipids Health Dis..

[31] Ixtaina V.Y., Martínez M.L., Spotorno V., Mateo C.M., Maestri D.M., Diehl B.W.K., Nolasco S.M., Tomás M.C. (2011). Characterization of chia seed oils obtained by pressing and solvent extraction. J. Food Compos. Anal..

[32] Dickinson E., Chen J. (1999). Heat-set whey protein emulsion gels: Role of active and inactive filler particles. J. Disper. Sci. Technol..

[33] Geremias-Andrade I.M., Souki N.P.D.B.G., Moraes I.C.F., Pinho S.C. (2017). Rheological and mechanical characterization of curcumin-loaded emulsion-filled gels produced with whey protein isolate and xanthan gum. LWT Food Sci. Technol..

[34] Herrero A.M., Carmona P., Pintado T., Jiménez-Colmenero F., Ruíz-Capillas C. (2011). Infrared spectroscopic analysis of structural features and interactions in olive oil-in-water emulsions stabilized with soy protein. Food Res. Int..

[35] Lee S.H., Lefévre T., Subirade M., Paquin P. (2007). Changes and roles of secondary structures of whey protein for the formation of protein membrane at soy oil/water interface under high-pressure homogenization. J. Agric. Food Chem..

[36] Alix A.J.P., Pedanou G., Berjot M. (1988). Fast determination of the quantitative secondary structure of proteins by using some parameters of the Raman amide I band. J. Mol. Struct..

[37] Niu F., Niu D., Zhang H., Chang C., Gu L., Su Y., Yang Y. (2016). Ovalbumin/gum Arabic-stabilized emulsion: Rheology, emulsion characteristics, and Raman spectroscopic study. Food Hydrocoll..

[38] AOAC (2005). Official Method of Analysis.

[39] Bligh E.G., Dyer W.J. (1959). A rapid method for total lipid extraction and purification. Can. J. Biochem. Physiol..

